# An inventory of coastal freshwater fishes from Amapá highlighting the occurrence of eight new records for Brazil

**DOI:** 10.3897/zookeys.606.9297

**Published:** 2016-07-21

**Authors:** Bruno F. Melo, Ricardo C. Benine, Ricardo Britzke, Cecile S. Gama, Claudio Oliveira

**Affiliations:** 1 Departamento de Morfologia, Instituto de Biociências, Universidade Estadual Paulista, Rubião Jr. s/n. 18618-689, Botucatu, São Paulo, Brazil; 2 Department of Vertebrate Zoology, National Museum of Natural History, Smithsonian Institution, Washington DC, USA; 3 Departamento de Zoologia, Instituto de Biociências, Universidade Estadual Paulista, Rubião Jr. s/n. 18618-689, Botucatu, São Paulo, Brazil; 4 Laboratorio de Acuacultura, Facultad de Ciencias Agropecuarias, Universidad Técnica de Machala, Av. Panamericana Km. 4½ Vía Machala-Pasaje, 070150, Machala, El Oro, Ecuador; 5 Instituto de Pesquisas Científicas e Tecnológicas do Estado do Amapá, Av. Feliciano Coelho, 1509, Trem, 68900-260, Macapá, Amapá, Brazil

**Keywords:** Amazon, French Guyana, Neotropical, South America, Teleostei

## Abstract

The Amazon Basin occupies a vast portion of northern South America and contains some of the highest species richness in the world. The northern Brazilian state of Amapá is delimited by the Amazonas River to the south, the Oyapock River to the northern boundary with French Guyana, and the Atlantic northeastern coast to Amazon estuary. Despite several expeditions to the Amazon in recent decades, little is known about the freshwater ichthyofauna from Amapá, with records limited to local inventories and species descriptions. This paper presents a compilation of the freshwater fish diversity sampled in fifteen sites covering two major Amapá ecoregions during the dry season of 2015. 120 species representing eight orders and 40 families are reported upon in this work. Eight species appear for the first time in the Brazilian territory providing new information for future conservation status evaluations.

## Introduction

At the northern limit of Brazil, the state of Amapá occupies the lower portion of the Amazon River basin at the border between Brazil, French Guyana, and Suriname. With more than 14 million hectares, ca. 90% of its native surface is still intact and ca. 73% is legally protected as either federal/state conservation units or indigenous territories (Bernard 2008). As example, the Tumucumaque National Park is the largest Brazilian protected unit and the largest continuous tropical forest national park in the world (Bernard 2008). Consequently, Amapá harbors a striking portion of the Neotropical fauna and flora in the Guiana Shield. That region along with the adjacent coastal ecosystems certainly needs further biological research.

**Figure 1.**
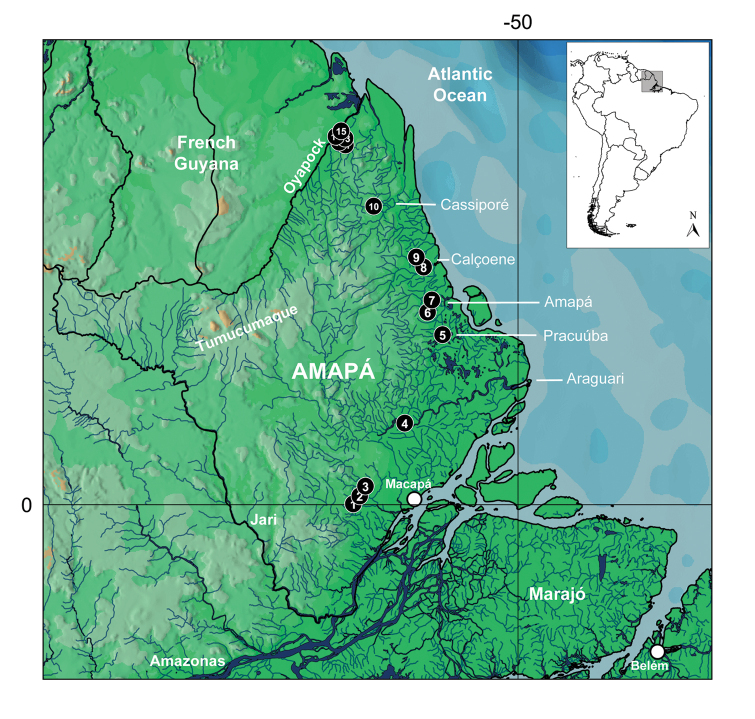
Map of Amapá in northern Brazil, lower Amazon basin showing each collecting site (black circles). Numbers match those in Table 1.

Amapá includes three major Neotropical freshwater ecoregions (sensu Abell et al. 2008). The first is the Guianas containing the Oyapock River that has its headwaters in the Tumucumaque National Park. The second is the Amazonas Guiana Shield containing the Rio Jari and Rio Iratapuru, and the upper portions of the Rio Araguari basin including the large Rio Amapari. Third ecoregion is the Amazonas Estuary and Coastal Drainages that contains small rivers reaching the lower Amazonas such as Rio Cajari and Rio Preto as well as several independent coastal rivers reaching the east coast, including the middle/lower Rio Araguari, Rio Flexal, Rio Amapá Grande and its associated lakes, Rio Calçoene, Rio Cunani, Rio Cassiporé and Rio Uaçá (Fig. 1) (Jégu and Keith 1999).

**Table 1.** Sampled localities in the Amapá and their placement in Figure 1.

Site	Locality	Major drainage	Municipality	Coordinates	Ecoregion
1	Rio Preto	Rio Amazonas	Mazagão	00°00'34.6"S 51°40'11.3"W	Amazonas Estuary and Coastal Drainages
2	Igarapé do Bispo	Rio Amazonas	Mazagão	00°01'45.5"N 51°38'05.6"W	Amazonas Estuary and Coastal Drainages
3	Unnamed lagoon	Rio Amazonas	Mazagão	00°05'11.3"N 51°37'58.1"W	Amazonas Estuary and Coastal Drainages
4	Unnamed stream	Rio Araguari	Ferreira Gomes	00°50'44.7"N 51°10'15.7"W	Amazonas Estuary and Coastal Drainages
5	Lago Pracuúba	Rio Amapá	Pracuúba	01°44'49.8"N 50°46'59.3"W	Amazonas Estuary and Coastal Drainages
6	Igarapé Balneário St. Bárbara	Rio Amapá	Amapá	02°03'42.8"N 50°54'15.1"W	Amazonas Estuary and Coastal Drainages
7	Igarapé Balneário Raso	Rio Amapá	Amapá	02°05'25.6"N 50°53'19.8"W	Amazonas Estuary and Coastal Drainages
8	Igarapé Pau Pintado	Rio Calçoene	Calçoene	02°28'47.7"N 50°58'47.2"W	Amazonas Estuary and Coastal Drainages
9	Igarapé Asa Aberta	Rio Calçoene	Calçoene	02°31'08.9"N 51°00'52.9"W	Amazonas Estuary and Coastal Drainages
10	Igarapé Faz. Campo Alegre	Rio Cassiporé	Oiapoque	03°04'49.7"N 51°28'50.7"W	Amazonas Estuary and Coastal Drainages
11	Igarapé do Quatorze	Oyapock River	Oiapoque	03°45'10.4"N 51°46'57.3"W	Guianas
12	Rio Pantanari	Oyapock River	Oiapoque	03°48'47.6"N 51°48'31.6"W	Guianas
13	Igarapé Cortiço	Oyapock River	Oiapoque	03°49'05.5"N 51°47'21.4"W	Guianas
14	Lagoa Mr. Rona	Oyapock River	Oiapoque	03°50'31.5"N 51°50'28.7"W	Guianas
15	Oyapock River	Oyapock River	Oiapoque	03°50'33.5"N 51°50'25.7"W	Guianas

Checklists of freshwater fishes have been continually published for French Guyana (Planquette et al. 1996; Keith et al. 2000; Le Bail et al. 2000, 2012), Suriname (Mol et al. 2012) and the Guiana Shield in general (Vari et al. 2009; Sidlauskas and Vari 2012). Likewise, biogeographic questions have been addressed to that region (Jégu and Keith 1999; Lujan and Armbruster 2011) as well as species descriptions (e.g. Jégu and Santos 1990; Vari 1992; Zarske and Géry 1998; Lucena and Gama 2007; Ottoni et. al. 2012). However, the diversity and composition of freshwater fishes from the Amapá and consequently the eastern limit of the Guiana Shield are limited to a concise and well-sampled inventory of fishes from the Tumucumaque National Park (Gama 2008) that is situated in the Amazonas Guiana Shield ecoregion (upper Oyapock River). Our aim is to present a survey of the ichthyofauna from Amapá sampled in the other two ecoregions: the Guianas (lower Oyapock River) and the Amazonas Estuary and Coastal Drainages. New records of freshwater fishes for Brazil should contribute to our understanding of the Neotropical ichthyofauna as well as to future evaluations of their conservation status.

## Material and methods

**Figure 2.**
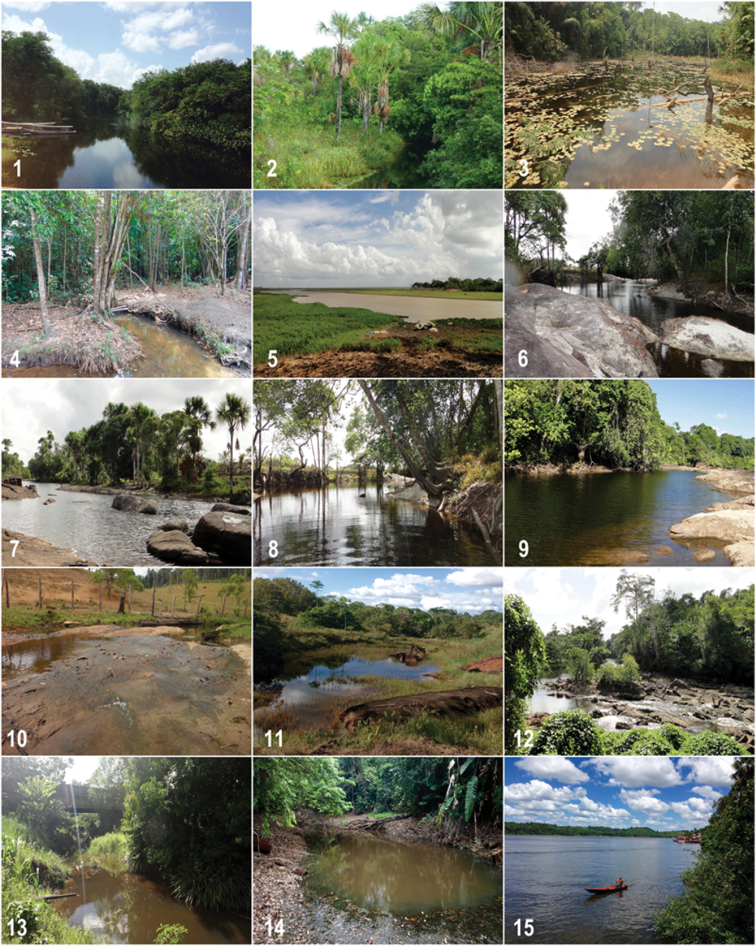
Sampled localities in the Amapá, northern Brazil. Numbers follow Table 1 and Fig. 1. Photographs by B.F. Melo and C. Oliveira. Photo 2 credits: Alan Kardec available at Google Earth.

Fifteen sites were sampled including small streams, river channels and large/lentic lagoons covering two ecoregions from Abell et al. (2008): Guianas (lower Oyapock River) and Amazonas Estuary and Coastal Drainages (Table 1; Fig. 1). Collection sites (Fig. 2) cover variable environments including small streams, rivers and lagoons and are near the main state road BR-156, which connects Laranjal do Jari to Oiapoque municipalities. Mazagão, Ferreira Gomes, Tartarugalzinho, Amapá and Calçoene are the intervening cities. Sampling efforts occurred during dry season in September and late November/early December 2015, mostly during daylight. Specimen collection involved tows of dipnets along the vegetated margins, a 5-meter bottom trawl and cast-netting in large streams or lagoons. Collection in the Lago Pracuúba comprised gillnets blocking fish passage overnight.

Specimens were anesthetized in a solution of clove oil, preserved in 96% ethanol (for molecular studies) or fixed in 10% formalin solution (for morphological studies) and then preserved in 70% ethanol. Muscle tissues were collected and preserved in 96% ethanol. Vouchers are deposited in the Laboratório de Biologia e Genética de Peixes at the Universidade Estadual Paulista, Botucatu, São Paulo, Brazil (LBP). Expeditions had permission to collect wild species in Brazil under ICMBio license number 13.843-1 to Claudio Oliveira. Species identifications to the lowest taxonomic level were conducted consulting the taxonomic literature and identification keys (Géry 1977; Planquette et al. 1996; Keith et al. 2000; Le Bail et al. 2000; Lucena and Malabarba 2010; Sidlauskas and Vari 2012; Peixoto et al. 2015) and updated with Eschmeyer et al. (2016). Classification follows the current phylogenetic arrangement of bony fishes (Betancur-R et al. 2014). We used checklists (Reis et al. 2003; Le Bail et al. 2012), SpeciesLink (http://www.splink.org.br/) and FishNet2 (http://www.fishnet2.net/) to search previous records of the species listed herein.

## Results and discussion

The fish survey in the freshwater rivers of Amapá resulted in 120 species representing eight orders and 40 fish families that range from obligate freshwater to marine organisms (Table 2). Orders comprising the highest percentage of species richness were Characiformes (55%), Cichliformes (16.6%), Siluriformes (14.1%) and Gymnotiformes (7.5%) as expected in Neotropical freshwaters (e.g. Langeani et al. 2007; Vari et al. 2009; Polaz et al. 2014). These four orders represent 93.3% of the total species richness. Clupeiformes (*Anchovia*), Cyprinodontiformes (*Fluviphylax* and *Melanorivulus*), Gobiiformes (*Dormitator*), Beloniformes (*Potamorrhaphis*), Sciaenidae (*Micropogonias*) and Polycentridae (*Monocirrhus* and *Polycentrus*) complete the list with one or two species each. The most highly represented family was Characidae (25% of total species), followed by Cichlidae (16.6%) and Loricariidae (6.6%). Previous fish survey from Tumucumaque National Park listed 207 species from five expeditions (Gama 2008) and found a new species record in the Brazilian territory, the cichlid *Cleithracara maronii*. It is noteworthy, however, that some species are common in both lists despite sampling distinct ecoregions (e.g. *Ageneiosus inermis*, *Apistogramma gossei*, *Bryconops caudomaculatus*, *Leporinus gossei*, *Microsternarchus bilineatus*).

**Table 2.** List of freshwater fish species collected in the state of Amapá, northern Brazil.

CLASS/ORDER/FAMILY/SPECIES	
CLASS ACTINOPTERYGII	
CLUPEIFORMES	
Engraulidae	
Anchovia aff. surinamensis (Bleeker, 1866)	LBP 20632
CHARACIFORMES	
Curimatidae	
*Curimata cyprinoides* (Linnaeus, 1766)	LBP 20629, LBP 20634, LBP 20645
*Curimatopsis* sp.	LBP 20480, LBP 21011, LBP 21031, LBP 21052, LBP 21079, LBP 21113, LBP 21191, LBP 21212, LBP 21240
*Cyphocharax gouldingi* Vari, 1992	LBP 21032, LBP 21078, LBP 21133, LBP 21189, LBP 21211
*Cyphocharax helleri* (Steindachner, 1910)	LBP 21140
Anostomidae	
*Leporinus friderici* (Bloch, 1794)	LBP 20633
*Leporinus gossei* Géry, Planquette & Le Bail, 1991	LBP 20626, LBP 21114
*Leporinus nijsseni* Garavello, 1990	LBP 21163
*Leporinus parae* Eigenmann, 1908	LBP 20653
*Schizodon fasciatus* Spix & Agassiz, 1829	LBP 20656
Chilodontidae	
*Chilodus zunevei* Puyo, 1946	LBP 21129, LBP 21137, LBP 21177
Crenuchidae	
*Characidium zebra* Eigenmann, 1909	LBP 21016, LBP 21033, LBP 21051, LBP 21082, LBP 21149, LBP 21161, LBP 21195, LBP 21210
*Crenuchus spilurus* Günther, 1863	LBP 21107, LBP 21237
Melanocharacidium cf. blennioides (Eigenmann, 1909)	LBP 21185
*Melanocharacidium dispilomma* Buckup, 1993	LBP 21160
*Microcharacidium eleotrioides* (Géry, 1960)	LBP 21159, LBP 21190
Hemiodontidae	
*Hemiodus gracilis* Günther, 1864	LBP 21186
*Hemiodus quadrimaculatus* Pellegrin, 1908	LBP 21151
Characidae	
*Astyanax bimaculatus* (Linnaeus, 1758)	LBP 20637, LBP 21004, LBP 21008, LBP 21080, LBP 21125, LBP 21167, LBP 21234
*Astyanax* sp.	LBP 21005, LBP 21024, LBP 21026
*Bryconamericus* sp.	LBP 21171
*Charax niger* Lucena, 1989	LBP 21086, LBP 21217
*Hemigrammus boesemani* Géry, 1959	LBP 20484, LBP 20983, LBP 21099, LBP 21112, LBP 21128, LBP 21200, LBP 21225, LBP 21250, LBP 21589
*Hemigrammus levis* Durbin, 1908	LBP 20983, LBP 21006, LBP 21027, LBP 21043, LBP 21222
*Hemigrammus ocellifer* (Steindachner, 1882)	LBP 21069, LBP 21100, LBP 21244
*Hemigrammus rodwayi* Durbin, 1909	LBP 21226
Hemigrammus cf. schmardae (Steindachner, 1882)	LBP 21022, LBP 21045, LBP 21201, LBP 21224, LBP 21246
*Hemigrammus unilineatus* (Gill, 1858)	LBP 21094
*Hemigrammus* sp.	LBP 21001, LBP 21025, LBP 21046, LBP 21047, LBP 21068, LBP 21071, LBP 21227
*Hyphessobrycon copelandi* Durbin, 1908	LBP 21036, LBP 21064, LBP 21103, LBP 21138, LBP 21241
*Hyphessobrycon* sp.	LBP 20483, LBP 21002, LBP 21003, LBP 21101, LBP 21196, LBP 21199, LBP 21221
*Jupiaba abramoides* (Eigenmann, 1909)	LBP 21095
*Jupiaba keithi* (Géry, Planquette & Le Bail, 1996)	LBP 21122, LBP 21169
*Jupiaba meunieri* (Géry, Planquette & Le Bail, 1996)	LBP 21007
*Jupiaba ocellata* (Géry, Planquette & Le Bail, 1996)	LBP 21088
*Jupiaba* sp.	LBP 21247
*Moenkhausia chrysargyrea* (Günther, 1864)	LBP 21070, LBP 21097, LBP 21121, LBP 21146
*Moenkhausia collettii* (Steindachner, 1882)	LBP 21010, LBP 21044, LBP 21066, LBP 21096, LBP 21198, LBP 21223, LBP 21248
*Moenkhausia georgiae* Géry, 1965	LBP 21093
*Moenkhausia gracilima* (Eigenmann, 1908)	LBP 21243
*Moenkhausia lepidura* (Kner, 1858)	LBP 21085, LBP 21145
*Moenkhausia oligolepis* (Günther, 1864)	LBP 21077, LBP 21124, LBP 21139
*Moenkhausia surinamensis* Géry, 1965	LBP 21009, LBP 21102, LBP 21245
*Moenkhausia* sp.	LBP 21098, LBP 21249
*Odontostilbe gracilis* (Géry, 1960)	LBP 21228
*Phenacogaster wayana* Le Bail & Lucena, 2010	LBP 21218
*Poptella brevispina* Reis, 1989	LBP 21014, LBP 21056, LBP 21104, LBP 21127, LBP 21141, LBP 21168, LBP 21194, LBP 21220
*Pristella maxillaris* (Ulrey, 1894)	LBP 21037
Acestrorhynchidae	
*Acestrorhynchus falcatus* (Bloch, 1794)	LBP 21173
*Acestrorhynchus microlepis* (Schomburgk, 1841)	LBP 21142
Erythrinidae	
*Hoplerythrinus unitaeniatus* (Agassiz, 1829)	LBP 21209
*Hoplias malabaricus* (Bloch, 1794)	LBP 21084, LBP 21110, LBP 21115, LBP 21175, LBP 21233
Lebiasinidae	
Copella aff. arnoldi (Regan, 1912)	LBP 20982
*Copella carsevennensis* (Regan, 1912)	LBP 21111, LBP 21131, LBP 21148, LBP 21162
*Nannostomus beckfordi* Günther, 1872	LBP 20477, LBP 20478, LBP 20981, LBP 21023, LBP 21034, LBP 21055, LBP 21091, LBP 21192, LBP 21216, LBP 21238
*Nannostomus bifasciatus* Hoedeman, 1954	LBP 21590
*Pyrrhulina filamentosa* Valenciennes, 1847	LBP 21015, LBP 21030, LBP 21054, LBP 21083, LBP 21193, LBP 21215, LBP 21239
Iguanodectidae	
*Bryconops affinis* (Günther, 1864)	LBP 21050, LBP 21132
*Bryconops caudomaculatus* (Günther, 1864)	LBP 20979, LBP 21089, LBP 21178, LBP 21219, LBP 21242
*Bryconops melanurus* (Bloch, 1794)	LBP 21143, LBP 21170
*Bryconops* sp.	LBP 20998, LBP 21013, LBP 21035
Serrasalmidae	
*Acnodon oligacanthus* (Müller & Troschel, 1844)	LBP 21012
*Metynnis lippincottianus* (Cope, 1870)	LBP 20647, LBP 20648, LBP 20999, LBP 21057, LBP 21172, LBP 21213
*Mylesinus paraschomburgkii* Jégu, Santos & Ferreira, 1989	LBP 20628
*Myleus ternetzi* (Norman, 1929)	LBP 20980, LBP 21144
*Pygocentrus nattereri* Kner, 1858	LBP 20651
*Serrasalmus humeralis* Valenciennes, 1850	LBP 20649, LBP 20650, LBP 20652, LBP 21187, LBP 21188, LBP 21214
SILURIFORMES	
Aspredinidae	
*Bunocephalus* sp.	LBP 21029
Callichthyidae	
*Megalechis thoracata* (Valenciennes, 1840)	LBP 21076
Loricariidae	
*Ancistrus* sp.	LBP 21147, LBP 21164
*Curculionichthys* sp.	LBP 21166
*Farlowella reticulata* Boeseman, 1971	LBP 21208, LBP 21230
*Guyanancistrus brevispinis* (Heitmans, Nijssen & Isbrücker, 1983)	LBP 21165, LBP 21183
*Hypostomus plecostomus* (Linnaeus, 1758)	LBP 20644
*Hypostomus ventromaculatus* Boeseman, 1968	LBP 20635
*Lithoxus* sp.	LBP 21184
*Loricaria cataphracta* Linnaeus, 1758	LBP 20636
Heptapteridae	
*Heptapterus bleekeri* Boeseman, 1953	LBP 21181
*Rhamdia quelen* (Quoy & Gaimard, 1824)	LBP 21072
Pimelodidae	
*Brachyplatystoma filamentosum* (Lichtenstein, 1819)	LBP 20630
*Hypophthalmus edentatus* Spix & Agassiz, 1829	LBP 20654
*Pimelodus* sp.	LBP 20627
Auchenipteridae	
*Ageneiosus inermis* (Linnaeus, 1766)	LBP 20655
*Trachelyopterus coriaceus* Valenciennes, 1840	LBP 20988
GYMNOTIFORMES	
Gymnotidae	
Gymnotus gr. anguillaris Hoedeman, 1962	LBP 21081, LBP 21156
Sternopygidae	
*Eigenmannia antonioi* Peixoto, Dutra & Wosiacki, 2015	LBP 21117, LBP 21182
*Eigenmannia waiwai* Peixoto, Dutra & Wosiacki, 2015	LBP 21118, LBP 21155
*Steatogenys elegans* (Steindachner, 1880)	LBP 20997
Apteronotidae	
*Apteronotus bonapartii* (Castelnau, 1855)	LBP 21591
Ramphichthyidae	
*Gymnorhamphichthys rondoni* (Miranda Ribeiro, 1920)	LBP 21232
*Rhamphichthys rostratus* (Linnaeus, 1766)	LBP 21120, LBP 21154
Hypopomidae	
*Brachyhypopomus pinnicaudatus* (Hopkins, 1991)	LBP 21130
*Microsternarchus bilineatus* Fernández-Yépez, 1968	LBP 20996, LBP 21053, LBP 21235
GOBIIFORMES	
Gobiidae	
*Dormitator maculatus* (Bloch, 1792)	LBP 21090
SUBSERIES OVALENTARIAE	
Polycentridae	
*Polycentrus schomburgkii* Müller & Troschell, 1849	LBP 21039, LBP 21109
*Monocirrhus polyacanthus* Heckel, 1840	LBP 20989, LBP 21028, LBP 21202
CICHLIFORMES	
Cichlidae	
*Acaronia nassa* (Heckel, 1840)	LBP 20985, LBP 21048
Aequidens gr. tetramerus (Heckel, 1840)	LBP 20994, LBP 21136, LBP 21231
*Apistogramma agassizii* (Steindachner, 1875)	LBP 20479
*Apistogramma gossei* Kullander, 1982	LBP 20993, LBP 21040, LBP 21062, LBP 21092, LBP 21236
*Apistogramma* sp. ‘Amapá’	LBP 21157
*Cichla monoculus* Spix & Agassiz, 1831	LBP 20625
*Cichla* sp.	LBP 21180, LBP 21205
Crenicichla cf. multispinosa Pellegrin, 1903	LBP 20991, LBP 21158
*Crenicichla saxatilis* (Linnaeus, 1758)	LBP 21038, LBP 21073, LBP 21207
*Geophagus surinamensis* (Bloch, 1791)	LBP 20638, LBP 20986, LBP 21074
*Guianacara geayi* (Pellegrin, 1902)	LBP 21116, LBP 21152
Heros cf. efasciatus Heckel, 1840	LBP 20646, LBP 20995, LBP 21042
*Hypselecara temporalis* (Günther, 1862)	LBP 21061, LBP 21067, LBP 21075, LBP 21135
Krobia aff. guianensis (Regan, 1905)	LBP 21017, LBP 21063, LBP 21108, LBP 21123, LBP 21153, LBP 21179, LBP 21203
*Laetacara flamannellus* Ottoni, Bragança, Amorim & Gama, 2012	LBP 21049
*Mesonauta guyanae* Schindler, 1998	LBP 20992, LBP 21018, LBP 21041, LBP 21059, LBP 21087, LBP 21119, LBP 21176, LBP 21204
*Nannacara aureocephalus* Allgayer, 1983	LBP 21065, LBP 21126
*Retroculus septentrionalis* Gosse, 1971	LBP 21019
*Satanoperca jurupari* (Heckel, 1840)	LBP 21058, LBP 21174
*Satanoperca rhynchitis* Kullander, 2012	LBP 21020
CYPRINODONTIFORMES	
Rivulidae	
*Melanorivulus schuncki* (Costa & De Luca, 2011)	LBP 20427, LBP 20987, LBP 21134, LBP 21197, LBP 21229, LBP 21251
Poeciliidae	
*Fluviphylax palikur* Costa & Le Bail, 1999	LBP 20481, LBP 20984, LBP 21021
BELONIFORMES	
Belonidae	
*Potamorrhaphis guianensis* (Jardine, 1843)	LBP 20990, LBP 21060, LBP 21206
SERIES PERCOMORPHARIA	
Sciaenidae	
*Micropogonias furnieri* (Desmarest, 1823)	LBP 20631

**Figure 3.**
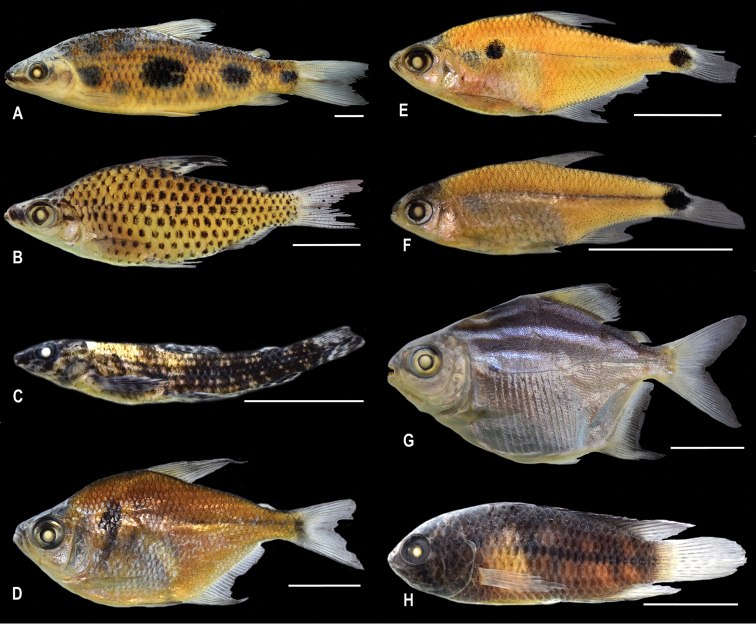
Species reported for the first time in Brazil. **a** *Leporinus gossei*, LBP 21114, Oyapock River **b** *Chilodus zunevei*, LBP 21129, Oyapock River **c** Melanocharacidium cf. blennioides, LBP 21185, Rio Amapá **d** *Jupiaba keithi*, LBP 21122, Oyapock River **e** *Phenacogaster wayana*, LBP 21218, Rio Amapá **f** *Odontostilbe gracilis*, LBP 21228, Rio Amapá **g** *Acnodon oligacanthus*, LBP 21012, Rio Calçoene **h** *Nannacara aureocephalus*, LBP 21065, Oyapock River. Scale bars: 1 cm.

Eight species were identified from Brazilian territory previously thought to inhabit only the Guianas region. Herein, these species are formally recorded for Brazil and are *Leporinus gossei*, *Chilodus zunevei*, Melanocharacidium cf. blennioides, *Jupiaba keithi*, *Phenacogaster wayana*, *Odontostilbe gracilis*, *Acnodon oligacanthus* and *Nannacara aureocephalus* (Fig. 3). Among them, *Leporinus gossei*, *Chilodus zunevei* and *Nannacara aureocephalus* were recently reported to Oyapock River in the Brazil-French Guyana boundary (Le Bail et al. 2012). *Leporinus gossei* was described from Marowijne river (Géry et al. 1991) and subsequently reported to many rivers of French Guyana (Planquette et al. 1996; Sidlauskas and Vari 2012) including the Brazilian section of the Oyapock River reported herein. *Chilodus zunevei* is recorded for the northeastern Suriname along the coastal rivers of French Guyana to Oyapock (Sidlauskas and Vari 2012) and now in the Rio Amapá. *Melanocharacidium blenioides* was described from the Potaro River in Guyana and ranges from Guyana to French Guyana (Buckup 2003). Herein, we report the species to the Rio Amapá. *Jupiaba keithi* is recorded from the Marowijne and Mana rivers of French Guyana (Zanata 1997); we found *Jupiaba keithi* in the Oyapock River. The recently described *Phenacogaster wayana* from Corantijn to Approuague rivers (Lucena and Malabarba 2010) is formally reported to Rio Amapá in Brazil. *Odontostilbe gracilis* occurs in a few places of the French Guyana (Planquette et al. 1996); we collected only a single specimen in the Rio Amapá. *Acnodon oligacanthus* is known only from the Guianas (Jégu 2003) and we found several specimens in the Rio Calçoene. Finally, *Nannacara aureocephalus* occurs in the Approuague river in French Guyana (Kullander 2003) and we found nine specimens in the Oyapock River. These new records should be incorporated in future evaluations of Brazilian threatened species conducted by the Ministério do Meio Ambiente/IUCN.

The following species are known to occur in Brazil but are now first reported for coastal rivers of Amapá. *Leporinus parae* occurs in the lower Amazon basin, state of Pará, Brazil as well as in the Orinoco basin in Venezuela (Britski and Birindelli 2008). We collected *Leporinus parae* in the Lago Pracuúba, an oxbow lake adjacent to the Rio Amapá Grande, leading to the first formal record of the species to Amapá. *Jupiaba abramoides* was previously known from the Rio Negro and we have now extended its range to the Oyapock River. Specimens of *Curculionichthys*, small armored catfishes distributed mostly throughout La Plata, São Francisco, Tapajós and Xingu basins (Roxo et al. 2015) were found in the Rio Cassiporé and Rio Jari and represent an undescribed species.

Besides *Curculionichthys*, three other taxa represent putative undescribed species: *Curimatopsis* sp., a member of the cryptic lineage Curimatopsis aff. crypticus (Melo et al. 2016), *Lithoxus* sp. from Rio Amapá and *Apistogramma* sp. ‘Amapá’ from the Oyapock River, commonly misidentified as *Apistogramma gossei*. These specimens are subject of ongoing taxonomic investigation in our laboratory. Furthermore, some taxa could only be identified at genus level, such as *Astyanax* sp., *Bryconops* sp., *Bryconamericus* sp., *Bunocephalus* sp., *Hemigrammus* sp., *Hyphessobrycon* sp., *Pimelodus* sp., among others (Table 2), and additional studies will be necessary to further classify these specimens. Overall, our survey increases our knowledge on the freshwater ichthyofauna of Amapá and of northern Brazil and provides new information for future conservation status evaluation. However, as other regions of the state remain unexplored, future inventories will likely reveal additional fish species in that part of the Guiana Shield.
